# Acquired contractile ability in human endometrial stromal cells by passive loading of cyclic tensile stretch

**DOI:** 10.1038/s41598-020-65884-3

**Published:** 2020-06-02

**Authors:** Jeonghyun Kim, Takashi Ushida, Kevin Montagne, Yasushi Hirota, Osamu Yoshino, Takehiro Hiraoka, Yutaka Osuga, Katsuko S. Furuakwa

**Affiliations:** 1 Department of Bioengineering, Graduate School of Engineering, the University of Tokyo, Tokyo, Japan; 2 Department of Mechanical Engineering, Graduate School of Engineering, the University of Tokyo, Tokyo, Japan; 3 Department of Obstetrics and Gynecology, School of Medicine, the University of Tokyo, Tokyo, Japan; 4 Department of Obstetrics and Gynecology, School of Medicine, Kitasato University, Sagamihara, Japan

**Keywords:** Regenerative medicine, Tissue engineering, Dynamical systems, Multicellular systems, Biotechnology, Systems biology

## Abstract

The uterus plays an important and unique role during pregnancy and is a dynamic organ subjected to mechanical stimuli. It has been reported that infertility occurs when the peristalsis is prevented, although its mechanisms remain unknown. In this study, we found that mechanical strain mimicking the peristaltic motion of the uterine smooth muscle layer enabled the endometrial stromal cells to acquire contractility. In order to mimic the peristalsis induced by uterine smooth muscle cells, cyclic tensile stretch was applied to human endometrial stromal cells. The results showed that the strained cells exerted greater contractility in three-dimensional collagen gels in the presence of oxytocin, due to up-regulated alpha-smooth muscle actin expression via the cAMP signaling pathway. These *in vitro* findings underscore the plasticity of the endometrial stromal cell phenotype and suggest the possibility of acquired contractility by these cells *in vivo* and its potential contribution to uterine contractile activity. This phenomenon may be a typical example of how a tissue passively acquires new contractile functions under mechanical stimulation from a neighboring tissue, enabling it to support the adjacent tissue’s functions.

## Introduction

It is now widely known that mechanical stimuli applied to various cell types can trigger intracellular signaling events leading to physiological and pathological changes^1–5^. The uterus allows implantation of the embryo and regulates its growth by supplying nutrients from the mother’s body^6^. It is also known as a dynamic organ that is modulated by menstrual hormone changes during the menstrual cycle and pregnancy. The uterine wall consists of three layers, namely the endometrium, the myometrium, and the perimetrium^7,8^. While the inner layer of the endometrium is composed of epithelial cells and stromal cells, the thickest middle myometrial layer mainly consists of smooth muscle cells. The perimetrium is the thin outermost layer of connective tissue. The myometrium is known to show spontaneous contractile activity^9^, and undergoes remodeling by hyperplasia and hypertrophy during pregnancy^10^.

The non-pregnant uterus also shows a distinct activity called “endometrium movement” throughout the menstrual cycle, which is regulated by ovarian steroid hormones^11^. Furthermore, the endometrium wave is known to play a significant role during pregnancy in order to transport the fertilized egg/zygote through the utero-tubal cavities prior to implantation^12^. It has also been reported that infertility occurs when the mechanical stress induced by the endometrium wave is prevented^13^. Therefore, we believe that this mechanical stimulus from the myometrium has a crucial role in physiological functions of the endometrium, such as menstruation and pregnancy.

In this study, we hypothesized that the uterine peristalsis induced by uterine smooth muscle cells might affect the contractile ability of endometrial stromal cells, an important function of the uterus for pregnancy. The endometrial stromal cells have been thought to passively undergo strain stimulation under the contractile movement of uterine smooth muscle cells. However, we propose that stromal cells actually actively support the peristaltic movement of the uterus. It is possible that the existence of these mechanisms helps to make the uterine peristaltic movement, which plays an important role for implantation of fertilized eggs and pregnancy, more steady and reliable.

## Results

### Reorientation of hESCs after applying 7 days of uniaxial cyclic strain

**Figure 1:**
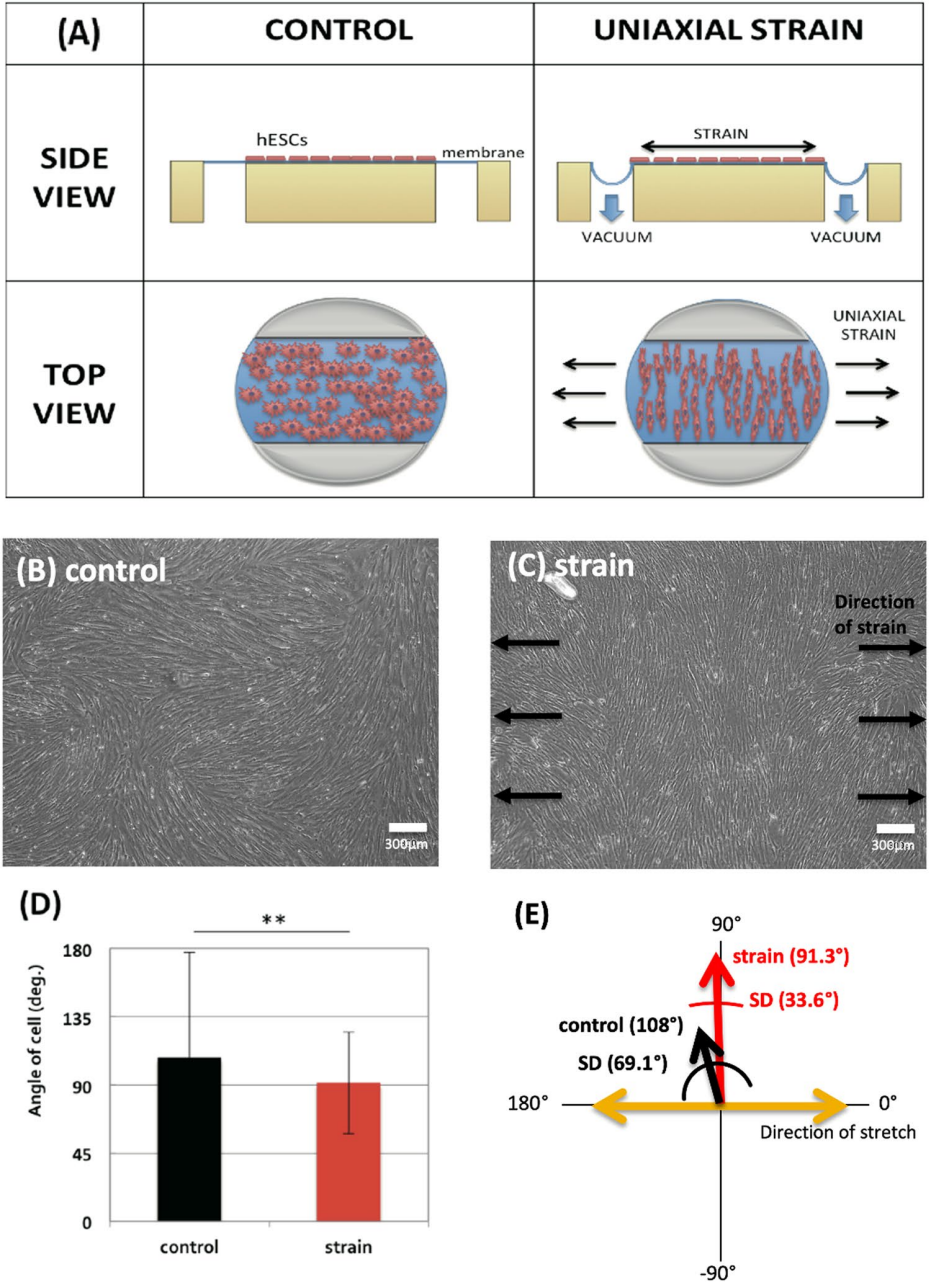
(**A**) Schematic view of the experimental setup showing the side view of the Flexcell tension system and the top view of the Flexcell plate. Microscope images of (**B**) control and (**C**) strained cells after applying 7 days of cyclic strain. The white bar indicates 300 μm. (**D**) Quantification of orientation changes in the strained cells. Graphs show the angle of cells (300 cells from 6 independent experiments). The bars represent the mean ± standard error deviation (p-value was obtained from F-test; *p < 0.05, **p < 0.005). (**E**) Schematic plot of the cell distribution. The red and black arrows represent the strained and control group, respectively.

In this study, we loaded 15% of uniaxial cyclic strain at 0.1 Hz to hESCs for 7 days, as shown in Fig. 1(A). In order to quantify the reorientation of hESCs after applying uniaxial cyclic strain for 7 days, we evaluated the changes in the cells’ angle from normal microscope images of control and strained cells as shown in Fig. 1(B),(C). In Fig. 1(D),(E), mean angles of cells (or mean direction of elongation) in control and strained hESCs were 108**°** (SD 69.1**°**) and 91.3**°** (SD 33.6**°**), respectively. The mean angle of strained hESCs compared to that of control cells was significantly different (*p* < 0.005). Moreover, the standard deviation of the angle in the strained cells was much smaller than that of the control sample. This indicates the angle of the cells in the strained hESCs was much more uniform compared to the more random orientation of the control hESCs. Hence, after 7 days, the strained cells reoriented perpendicularly to the direction of strain and became elongated while the control cells were randomly oriented.

### Uniaxial cyclic strain up-regulated α-SMA expression in hESCs

**Figure 2:**
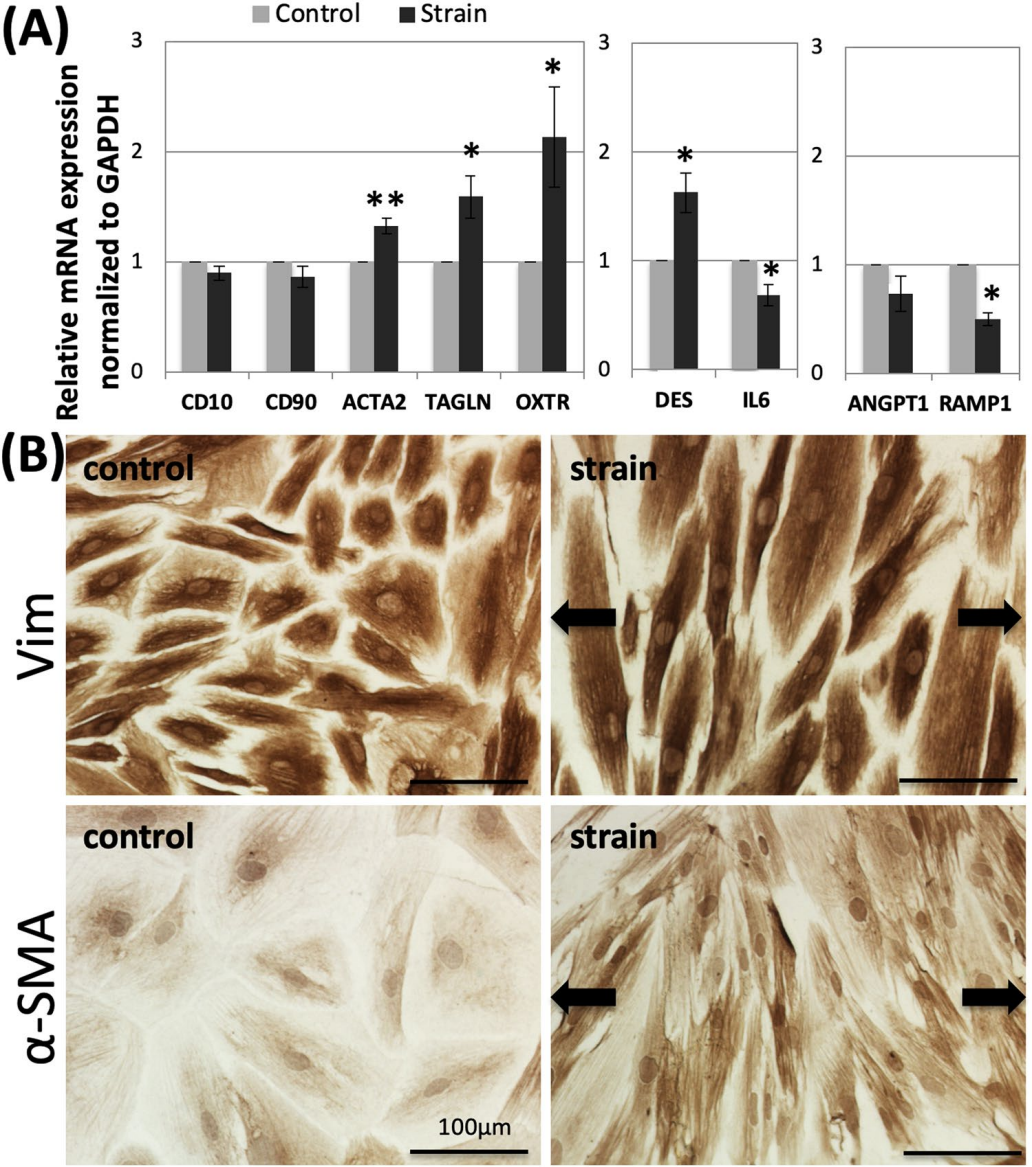
Promoted expressions of smooth muscle cell markers in hESCs after applying cyclic strain for 7 days, measured by real-time PCR and immunostaining. (**A**) mRNA expressions of endometrial stromal cell markers and smooth muscle cell markers in hESCs after applying cyclic strain for 7 days measured by real-time PCR. (*CD10, CD90, ACATA2, TAGLN, OXTR, DES*, *IL6, ANGPT1*, and *RAMP1*). Graphs show the fold change of mRNA expressions relative to *RPL32* mRNA normalized to the control mean (*n* = 6). The bars represent the mean ± standard error (p-value was obtained from Student’s *t*-test; *p < 0.05, **p < 0.005). (**B**) Immunostaining of Vimentin (Vim) and smooth muscle actin (α-SMA) in hESCs after loading cyclic strain for 7 days; Vim and α-SMA expression in control samples (left). Vim and α-SMA expression in strained samples (right). The arrows indicate the direction of cyclic strain. Scale bar = 100 μm.

As shown in Fig. 2(A), we analyzed by real-time PCR several stromal cell markers including *CD10* and *CD90* and uterine smooth muscle cell (SMC) markers such as *ACTA2* and *TAGLN*. Particularly, *ACTA2* plays a key role in the production of alpha-smooth muscle actin (α-SMA), which belongs to the actin protein family and is involved in cell contraction. Cyclic strain slightly decreased endometrial stromal cell marker expression (0.90-fold change for *CD10* and 0.86-fold change for *CD90*), but the changes were not significant. With regards to *ACTA2* and *TAGLN*, they were significantly up-regulated by cyclic strain (1.32-fold change for *ACTA2*, *p* < 0.005; and 1.59-fold change for *TAGLN, p* < 0.05). In the same manner as *ACTA2* and *TAGLN*, 7 days of cyclic strain significantly raised mRNA expression of the oxytocin receptor (*OXTR*), which is highly expressed in the myometrium to regulate uterine contraction (2.13-fold change for *OXTR, p* < 0.05).

In order to distinguish uterine SMCs and myofibroblasts, we also examined desmin (*DES*) and Interleukin 6 (*IL6*) expression, which are associated with myofibroblasts. Real-time PCR results also showed an up-regulation of *DES* mRNA expression (1.63-fold change, *p* < 0.05) and a decrease in *IL6* (0.68-fold change; p < 0.05) after application of cyclic strain. On the other hand, mRNA expressions of Angiopoietin 1 (*ANGPT1*) (0.73-fold change; *p* = 0.12) and Receptor activity modifying protein (*RAMP1*) (0.50-fold change, *p* < 0.005) were measured to distinguish uterine SMCs and vascular smooth muscle cells.

After applying cyclic strain for 7 days, immunostaining for vimentin (Vim) and α-SMA was carried out to check for changes in stromal cell and smooth muscle cell marker expression as shown in Fig. 2(B). As a result, there was an increase in the staining intensity of α-SMA in the strained cells while no significant change in the expression of the stromal cell marker was observed after applying cyclic strain.

### Cyclic strain increases cAMP production in hESC

**Figure 3:**
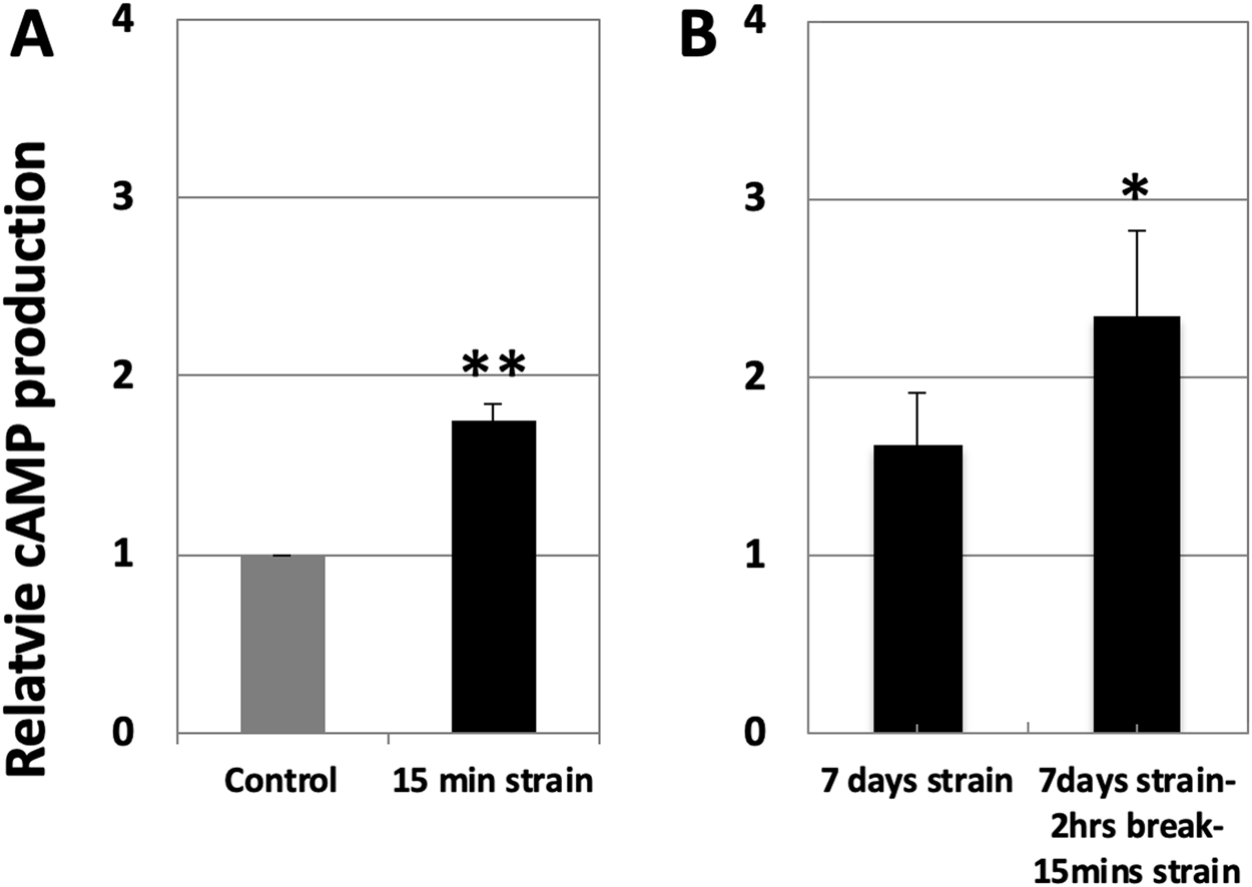
Relative cAMP production levels measured by the cyclic AMP EIA kit. (**A**) Applying cyclic strain for 15 mins significantly up-regulated cAMP production in hESCs. (**B**) cAMP levels were measured after 7 days of strain, followed by a 2-hour break for cAMP levels to stabilize, followed by an extra 15 mins of cyclic strain. 7 days of strain induced a non-significant increase in cAMP production, but adding a 2-hour break followed by an extra 15 mins significantly up-regulated cAMP levels. Graphs show the fold change of cAMP production levels relative to the amounts of DNA and normalized to the control mean. The bars represent the mean ± standard error (*n* = 4) (p-value was obtained from Student’s *t*-test; *p < 0.05, **p < 0.005).

In order to understand the effect of uniaxial cyclic strain on hESCs, we measured the level of cAMP in hESCs after applying strain. Firstly, cAMP concentrations after 15 mins of cyclic strain were measured. Figure 3(A) shows a transient and significant up-regulation in cAMP production (1.75-fold change; *p* < 0.005) in as little as 15 mins. In addition, Fig. 3(B) shows the levels of cAMP after 7 days of cyclic strain. There was a non-significant increase in cAMP production immediately after 7 days of strain (1.62-fold change; *p* = 0.07). We then performed 7 days of strain, followed by a 2-hour break for cAMP levels to stabilize, followed by an extra 15 mins of cyclic strain. After such a strain regimen, cAMP concentration was significantly up-regulated (2.35-fold change; *p* < 0.05).

### SQ22536 and H-89 inhibit the up-regulation of α-SMA expression by cyclic strain in hESCs

**Figure 4:**
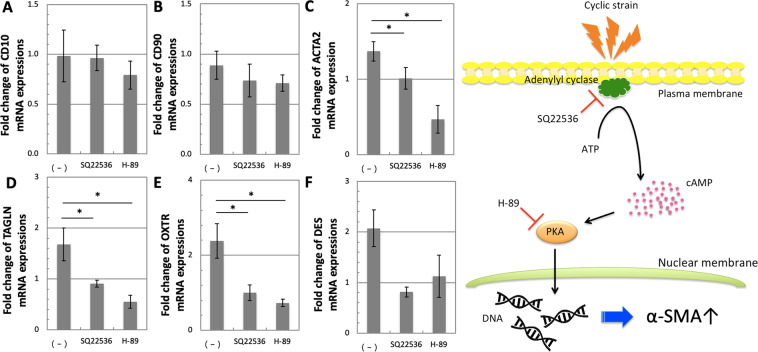
mRNA expressions measured by real-time PCR of (**A**) *CD10*, (**B**) *CD90*, (**C**) *ACTA2*, (**D**) *TAGLN*, (**E**) *OXTR*, and (**F**) *DES* in hESCs after applying cyclic strain in the presence or absence of the inhibitors SQ22536 and H-89. All the mRNA expressions were normalized to *RPL32* expression and further normalized to control values. Applying cyclic strain for 7 days non-significantly down-regulated both endometrial stromal cell markers and up-regulated the smooth muscle cell markers. While the non-significant down-regulation of *CD10* and *CD90* were unchanged by addition of SQ22536 and H-89, both inhibitors significantly inhibited the up-regulation of *ACTA2* and *OXTR*. The bars represent the mean fold change ± standard error between strained and control samples (*n* = 4) (p-values were obtained from ANOVA followed by Fisher’s LSD test; *p < 0.05). (**G**) Schematic diagram of the signaling pathway activated in hESCs in response to cyclic strain. Adenylyl cyclase located on the inner side of the plasma membrane converts ATP to intracellular cAMP. cAMP induced by cyclic strain then promoted SMa marker α-SMA expression, via adenylyl cyclase and PKA.

To determine whether the cAMP pathway is involved in the up-regulation of α-SMA expression under strain, we stretched hESCs in the presence or absence of the adenylyl cyclase inhibitor SQ22536 or the PKA inhibitor H-89. Figure 4(A)–(F) represent the fold changes in mRNA expression measured by real-time PCR in the presence or absence of inhibitors. As in the previous experiment, the cyclic strain did not significantly affect *CD10* or *CD90* expression in hESCs but significantly up-regulated *ACTA2* (1.37-fold change) and *TAGLN* (1.68-fold change) expression. By adding SQ22536, the up-regulation of *ACTA2, TAGLN*, and *OXTR* by cyclic strain was inhibited, with a respectively 1.01-, 0.91-, and 1.00-fold change in *ACTA2*, *TAGLN*, and *OXTR* expression. Moreover, the use of H-89 also showed an inhibiting effect on *ACTA2* (0.47-fold change), *TAGLN* (0.55-fold change), and *OXTR* (0.73-fold change) expression. Moreover, the SQ22536 and H-89 non-significantly suppressed the increase in *DES* (0.81- and 1.12-fold change, respectively) expression.

### Effect of oxytocin on the hESC-mediated collagen I gel contraction

**Figure 5:**
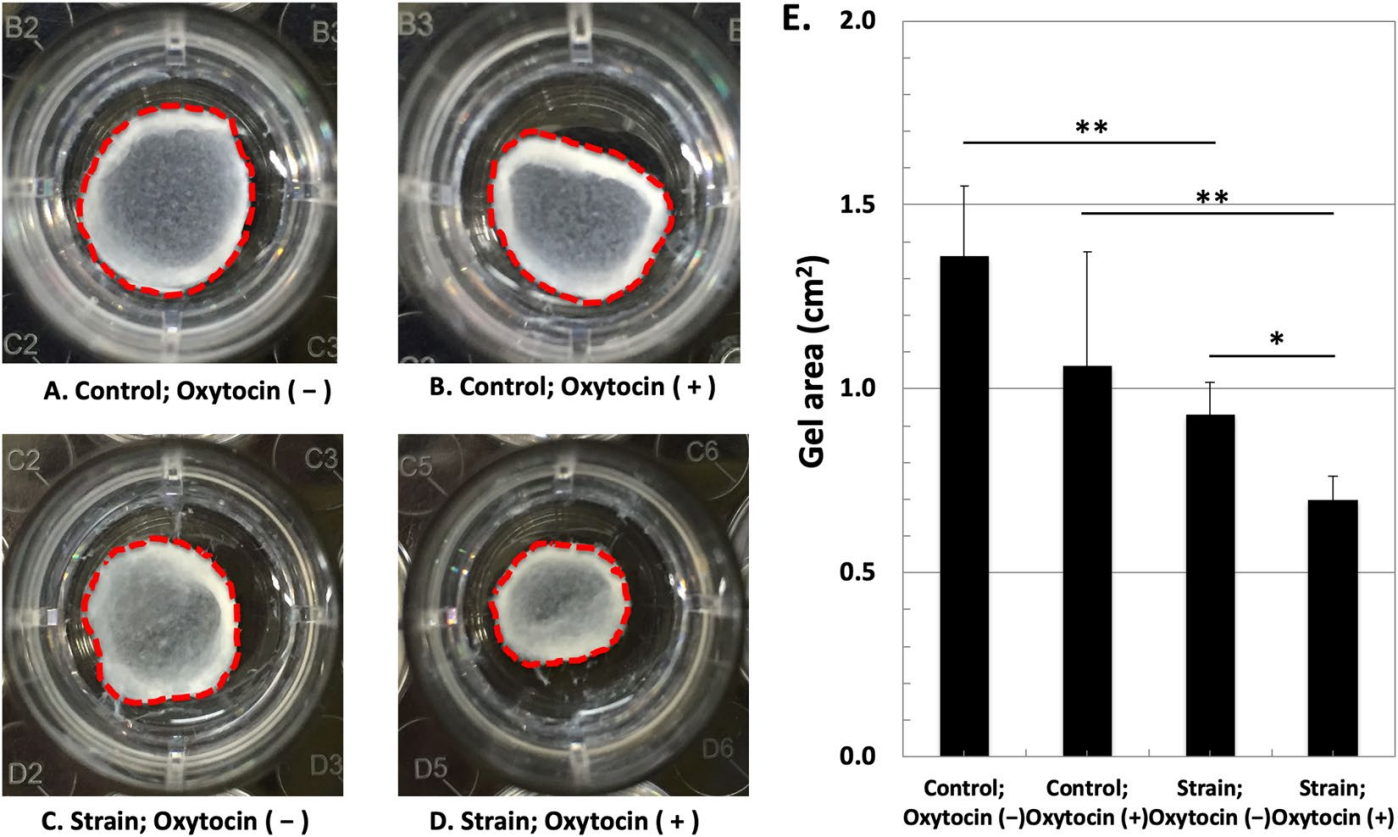
hESCs-mediated collagen I gel contraction after 7 days in response to oxytocin (10 nM). (**A**) control sample without oxytocin (**B**) control sample with oxytocin, (**C**) gel containing strained cells without oxytocin, and (**D**) gel containing strained cells with oxytocin. (E) The graph represents the gel area 7 days after release normalized to the initial area. The bars represent the mean ± standard error of gel area (*n* = 4) (p-value was obtained from ANOVA followed by Fisher’s LSD; *p < 0.05, **p < 0.005; the statistical analysis was performed using the original area measurement data).

After applying uniaxial cyclic strain to hESCs for 7 days, the strained cells were collected for a cell contraction assay to check the contractile ability of the cells seeded in a three-dimensional collagen gel. Before the stress in the gel was released by detaching the gels from the culture dish, the cells in the collagen I gel were treated with oxytocin (10 nM) to examine if in the presence of oxytocin, the strained cells exerted greater contractility, behaving like uterine SMC. Figure 5(A)–(D) show the samples 7 days after releasing the gels from the culture dish in the presence or absence of oxytocin. The surface areas of the samples were measured and quantified as shown in Fig. 5(E). The surface areas of the control samples with and without oxytocin shrank to 71.6% (1.36 cm^2^) and 55.9% (1.06 cm^2^), respectively. On the other hand, the strained cells showed a significant elevation in their contractile ability both in the absence and in the presence of oxytocin, as gels shrank to 48.9% (0.93 cm^2^) and 36.7% (0.70 cm^2^), respectively, of their initial surface area. In particular, while the strained cells showed greater contractility than the control cells (*p* < 0.005), the addition of oxytocin in the strained cells further enhanced their contractility (*p* < 0.05).

## Discussion

Mechanical stimulation is crucial to the proper function of many different organs like cartilage, blood vessels or the uterus^14,15^. For the uterus, which plays an important role during pregnancy and childbirth, the mechanical stress caused by the peristaltic movement of the fallopian tube due to the contraction of uterine smooth muscle cells promotes the migration of fertilized eggs^16^. In addition, infertility can also be caused by uterine fibroids that perturb the periodic uterine peristaltic movement induced by hormones^17^. Therefore, although the number of studies regarding mechanical stress responses in the uterus is still limited, the mechanical stress may play a role in homeostasis and pathogenesis of the uterus.

In this study, we proposed a new hypothesis regarding the response to mechanical stress in the uterine tissues with a hierarchical structure. The uterus has a three-layer structure consisting of, starting from the inside, epithelial cells, stromal cells, and smooth muscle cells. From our *in vitro* results, it is reasonable to imagine a similar mechanism *in vivo*, by which the peristaltic movement of the uterus caused by contraction of the outermost smooth muscle layer enhances the contractility of the inner stromal cells. We believe that such a mechanism might make the three-dimensional peristaltic movements of the uterus during pregnancy and childbirth more consistent and reliable.

In two-dimensional culture, the tensile stress that mimics the peristaltic movement of the myometrium is reported to regulate the biochemical function of stromal cells to support a the differentiation process of endometrium, decidualization^18^, but it is dubious whether the tensile stress is well loaded three-dimensionally on the stromal cells in the body. As shown in this study, acquisition of contractility by the stromal cells under strain may be an effective mechanism to transmit the tensile stress from the smooth muscle layer in the body. In other words, it is suspected that contraction by uterine smooth muscle cells is transmitted to the inner stromal cells, whereby each individual stromal cell can contract steadily thanks to the increased expression of proteins such as α-SMA and oxytocin. Considering the fact that, *in vivo*, infertility can occur when the uterus is unable to contract properly, these findings may represent a new mechanotransduction mechanism, by which contractility is transmitted from the outside to the adjacent inner cell layer.

It is possible that such a phenomenon occurs not only in the uterus, but also in blood vessels as reported in the literature. When the tensile stress is applied to vascular endothelial cells such as human umbilical cord endothelial cells (HUVEC), smooth muscle actin expression is dramatically increased^19–21^. It might imply that the vascular endothelial cells loaded with tensile stimulation may not differentiate into vascular smooth muscle cells, and individual vascular endothelial cells might have instead acquired the contractility under the tensile stimulation induced by mechanical activation of intracellular signaling pathways, thus potentially enhancing the efficiency of blood vessel contraction. Our experiments in hESCs have shown that the strain slightly increases the gene expression levels of SMC markers, *ACTA2*^22^ and *TAGLN*^23^. On the other hand, the immuno-staining data still showed strong staining levels of vimentin, a marker for stromal cells^24^, even with or without strain. Although Additionally, the α-SMA staining levels in ESC were extremely low compared to those in the SMC *in vivo*^24^. However, the strain did not apparently increase those of α-SMA in ESC even after stretching. Taking into consideration those results, it is reasonable to conclude that strain did not differentiate ESC into SMC, but made ESC acquire the ability to contract. This phenomenon might be similar to the response of HUVEC under strain.

In the field of cell and tissue engineering, mechanical stimuli have been identified as a significant factor to induce physiological changes by activating intracellular signaling pathways. While many researchers have reported on the effects and roles of mechanical stimuli in various cell models, studies of the uterus in response to mechanical stimuli are limited. There have been only a few studies reporting the effect of mechanical stretch on hESCs, particularly focusing on the expression of specific genes such as *IGFBP1* or interleukin-8 (*IL-8*), which are associated with decidualization or inflammatio^18,25^. In this study, we aimed to understand the effects of mechanical stimuli on the hESCs, particularly on their contractility.

We applied 15% of uniaxial cyclic strain to hESCs at 0.1 Hz for up to 7 days. First, application of uniaxial cyclic strain to hESCs induced rearrangement in the direction perpendicular to the strain axis while control cells were randomly distributed. Moreover, both real-time PCR and immunostaining showed that the cyclic strain induced an increase in the expression of α-SMA. As well as the up-regulation of α-SMA, real-time PCR results showed a significant up-regulation in the mRNA expression of oxytocin receptor (*OXTR*) after loading cyclic strain for 7 days, a gene which is highly expressed in the myometrium^26–28^. The oxytocin receptor, however, is also expressed in the endometrium and its expression varies during the non-pregnant cycle, depending on ovarian steroid hormones, such as progesterone and estrogen^29,30^. Kunz’s group showed that oxytocin increases the frequency of the endometrial wave^31^. Moreover, it is known that up-regulation of the oxytocin receptor before the onset of labor during pregnancy induces the production of prostaglandin F_2α_, which results in an increase in endometrial contraction. This study is the first to suggest that mechanical stimuli play a role in endometrial stromal cells in the acquisition of contractility, by up-regulating α-SMA and oxytocin receptor expression.

In addition, we showed the strained cells to be different from myofibroblasts. While there is a lack of desmin accumulated in myofibroblasts, the expression of *DES* is known to be relatively abundant in the myometrium^32,33^. Moreover, *IL6* production level is known to be elevated in myofibroblasts^34–36^. Application of cyclic strain for 7 days significantly induced the expression of *DES* and also down-regulated the expression of *IL6*, which indicates the strained hESCs were distinct from myofibroblasts. On the other hand, an essential marker abundantly expressed in vascular smooth muscle cells, *ANGPT1*, was non-significantly but slightly reduced by cyclic strain^37–40^. *RAMP1* mRNA expression, which is specific to vascular smooth muscle cells in uterine arteries, was significantly down-regulated by cyclic strain, indicating that the strained cells were distinct from vascular smooth muscle cells^41–43^.

In this study, a cAMP production assay was performed after loading strain on hESCs. cAMP is a secondary messenger produced from adenosine triphosphate (ATP) and is known to regulate endometrial stromal cells for decidualization during the menstrual cycle^44^. There is a report that addition of estradiol in uterine cells evoked an increase in cAMP levels, and the cAMP pathway via adenylyl cyclase is involved in this mechanism^45^. Moreover, the phenotype induced in bone marrow-derived MSCs by cAMP treatment suggests those cells could serve as a source of endometrial stem/progenitor cells^46^. Thus, cAMP has a significant regulatory role in the uterus just like hormones such as estrogen and progesterone. In this study, we therefore focused on the involvement of cAMP in response to cyclic strain.

Here we also report that cyclic strain up-regulated cAMP production in hESCs, implying that the cAMP signaling pathway may be involved in the up-regulation of α-SMA expression under stretch. Applying cyclic strain for as little as 15 mins induced the up-regulation of cAMP production in hESCs. After up-regulating α-SMA expression in hESCs under cyclic strain for 7 days, we also examined whether cAMP production was responsive to strain. Since cAMP production is usually transiently induced, cells strained for 7 days were subjected to a 2-hour break (static condition) to stabilize the level of cAMP, followed by 15 mins of cyclic strain. This resulted in a significant up-regulation of cAMP production, showing that stretch was able to induce cAMP production both before and after applying cyclic strain for 7 days.

In order to determine the importance of cAMP in the stretch-induced up-regulation of α-SMA expression, we carried out inhibitor tests using the adenylyl cyclase inhibitor SQ22536 and the PKA inhibitor H-89, since adenylyl cyclase regulates cAMP production while PKA is a well-known cAMP-dependent protein kinase^47,48^. Figure 4(G) schematically illustrates the signaling pathway activated in the hESCs under cyclic strain, as discussed in this study. Adenylyl cyclase is an enzyme located on the inner side of the plasma membrane and usually activated by G proteins. Activation of adenylyl cyclase under cyclic strain converts adenosine triphosphate (ATP) to cAMP, an intracellular second messenger. We showed that cyclic strain increased the cAMP production level in hESCs. This transient up-regulation of cAMP in hESCs by cyclic strain was consistent with previous studies using other cell models under mechanical stimuli such as cyclic strain or static compressive strain^49,50^. As suggested by the inhibitor tests, the increase in intracellular cAMP levels is essential for the up-regulation of α-SMA expression in hESCs by cyclic strain. Interestingly, the addition of H-89 led to a decrease in the mRNA expression of SMC markers and *OXTR* under cyclic strain. The result may imply that H-89 did not only specifically inhibit the cAMP signaling pathway, but also activated other signaling pathways which inhibit SMC markers and *OXTR* expression under strain. Further studies will be required to address this point.

The cell contraction assay using collagen gels was then carried out to measure the cells’ contractile ability. As a result, the samples strained for 7 days showed an increased contractile ability compared to control samples. Moreover, the contractility in the strained samples was significantly enhanced in the presence of oxytocin, indicating that the strained hESCs behaved like uterine smooth muscle cells. It has been reported that the enhanced contractile ability of ESC may help to minimize defects in an endometrial wound model and promote endometrial tissue repair *in vivo*^51^. In ruminants, the level of oxytocin receptor is known to increase during the diestrus phase, reach its maximum value during the proestrus phase, and then decline during the estrus phase^52,53^. Peristaltic patterns such as intrauterine pressure and strain have also been reported to change during the estrous cycle^54^. Up-regulation of *OXTR* mRNA expression induced by mechanical stimulation might contribute to stopping the bleeding during the menstrual cycle by strengthening the contractility of the endometrium in response to oxytocin. Although the endometrium is exposed to a dynamic environment induced by the myometrium, the effect of mechanical stimuli on the ESCs with regard to their contractility remained unknown. In this study, we suggested that the enhanced contractility in the strained cells was due to the up-regulation of α-SMA expression and the oxytocin receptor. While several studies have shown that biochemical stimulation using cytokines or platelet-derived growth factor (PDGF) increased the contractility in hESCs *in vitro*^51,55^, we are the first to report that mechanical stimuli also allowed endometrial stromal cells to acquire greater contractility while keeping their original cell phenotype. In other words, mechanical stimulation might help to control the dynamic and active functions of endometrial stromal cells. It has been reported that *OXTR* is not expressed in stromal cells *in vivo* by immuno-staining^27^ or *in situ* hybridization^56^. However, in this paper, we reported that strain up-regulated *OXTR* mRNA expression in stromal cells by real-time PCR, which enables more sensitive signal detection than immune-staining and *in situ* hybridization. However, our current studies have only been performed *in vitro*, and the relevance of our findings will be examined during further studies. Although there are limitations to direct extrapolation of *in vitro* result to the *in vivo* context, passive strain stimulation of stromal cells caused by uterus SMCs might trigger active stromal cell contraction.

In summary, we report that applying uniaxial cyclic strain significantly up-regulates the expression of α-SMA as well as cAMP production. Together, the results show that strained hESCs acquire greater contractility, thus behaving more like uterine smooth muscle cells. Furthermore, these findings may imply that contractile movements by the myometrium have a significant role in inducing endometrial stromal cells to acquire the ability to contract *in vivo* for physiological functions of the endometrium. This newly reported phenomenon might be a typical example of how a tissue passively acquires new contractile functions under mechanical stimulation from a neighboring tissue, enabling it to support the adjacent tissue’s functions.

## Methods

### Isolation and culture of hESCs

Endometrial biopsies were obtained from 38~48-year-old female patients who had regular menstrual cycles. Fresh human endometrial stromal cells were isolated and cultured as previously reported^57–59^. The purity of the cell source was greater than 98%^59^. This study was approved by the Institutional Review Board of the University of Tokyo in accordance with the Declaration of Helsinki, and each patient gave informed consent for sample collection. We cultured hESCs in DMEM/Ham’s F12 (Sigma) supplemented with 2.5% charcoal-stripped FBS (Funakoshi) and 1% of Antibiotic-Antimycotic (GIBCO) in a humidified incubator at 37 °C with 5% CO_2_. The culture medium was changed every 3 or 4 days. For inhibitor tests, we used the adenylyl cyclase inhibitor SQ22536 and the protein kinase A (PKA) inhibitor H-89 (both from Cayman Chemical). SQ22536 (100 μM) and H-89 (10 μM) were added to the cells’ culture medium just before loading cyclic strain.

### Uniaxial cyclic strain loading

In this study, we loaded cyclic strain using the Flexcell tension system (FX-4000; Flexcell International Corporation) placed in a humidified incubator at 37 °C with 5% CO_2_. The Flexcell is computer-operated and applies its strain by vacuum. In this study, 15% uniaxial strain was applied at 0.1 Hz as illustrated in Fig. 1. During application of the uniaxial cyclic strain, the culture medium was changed every 2 or 3 days. For control samples, hESCs were cultured in identical Flexcell plates but without strain.

### Measurement of cell reorientation under cyclic strain

In order to measure the cells’ orientation angles, we used normal microscope images (×5) taken from the center of Flexwell plate to cover a wide range of the sample, that is further to avoid sample vialing. By using ImageJ, a line was drawn from the bottom of the cell to the top of the cell along the cell’s major axis. After drawing the line, the angle between this line and the horizontal axis was measured using ImageJ. We selected 50 cells per each sample from 6 different experiments so as to select entirely in terms of area distribution.

### Real Time-PCR

To measure mRNA expression in the different samples from the Flexcell system, we carried out real-time PCR. After finishing loading cyclic strain, the cells were rinsed with PBS and immediately lysed with Trizol reagent (Invitrogen) before RNA extraction and cDNA synthesis using the ReverTra Ace qPCR RT Master Mix with gDNA Remover (Toyobo). *CD10* and *CD90* were used as endometrial stromal cell markers while *ACTA2* and transgelin *(TAGLN*) are highly expressed in SMC. Oxytocin receptor (*OXTR*) was also examined as a uterine smooth muscle cell marker while desmin (*DES*) and interleukin 6 (*IL6*) were utilized to distinguish uterine SMCs from myofibroblasts. Similarly, the expression of the vascular smooth muscle cell markers angiopoietin 1 (*ANGPT1*) and receptor activity modifying protein 1 (*RAMP1*) were also measured. All genes were normalized to *RPL32* expression and further normalized to the control samples. Primer sequences and amplicon sizes are listed in Table 1.

**Table 1:** Primer List.

Gene	Forward primer	Reverse primer	Amplicon size (bp)
*RPL32*	GCCCAAGATCGTCAAAAAGA	GTCAATGCCTCTGGGTTT	98
*CD10*	TCCACTGGAGATCAGCCTTT	TATCGGGAACTGGTCTCAGG	237
*CD90*	CTAGTGGACCAGAGCCTTCG	TGGAGTGCACACGTGTAGGT	235
*TAGLN*	AGGTCTGGCTGAAGAATGGC	TTCAAAGAGGTCAACAGTCTGG	199
*ACTA2*	CTGAGCGTGGCTATTCCTTC	TTCTCAAGGGAGGATGAGGA	133
*OXTR*	TTCTTCGTGCAGATGTGGAG	ACGAGTTCGTGGAAGAGGTG	149
*DES*	CTGAGCAAAGGGGTTCTGAG	TGGCAGAGGGTCTCTGTCTT	135
*IL6*	CACACAGACAGCCACTCACC	TTTTCTGCCAGTGCCTCTTT	139
*ANGPT1*	GAAGGGAACCGAGCCTATTC	GCTCTGTTTTCCTGCTGTCC	108
*RAMP1*	CCTCACCCAGTTCCAGGTAG	GAACCTGTCCACCTCTGCAT	157

### Immunostaining

After 7 days of cyclic strain, hESCs were immediately fixed with 4% paraformaldehyde. The fixed samples were permeabilized with 0.2% Triton-X 100 in PBS for 3 min and washed with PBS 3 times. Then non-specific binding was blocked with PBS containing 1% BSA before covering the cells with anti-α-SMA antibody (Abcam) at a 1/500 dilution for 1 hour at room temperature or overnight at 4**°**C. After revealing the antibody using the DAB peroxidase substrate kit (Vector Laboratories), the samples were mounted on glass slides for visualization and storage.

### Cyclic adenosine monophosphate (cAMP) measurement

The samples were collected and lysed with 0.1 M HCl. After centrifugation at 1,000 g the supernatant was decanted and stored at −80 °C until assay. Cyclic AMP concentrations were measured using the cyclic AMP EIA kit (Cayman Chemical) according to the manufacturer’s instructions. The absorbance at 412 nm was measured with an EnSpire Multimode Plate Reader (PerkinElmer). The cAMP concentrations were normalized to the DNA amounts quantified using the Quant-iT PicoGreen dsDNA Reagent and Kit (Invitrogen).

### Cell contraction assay

After the samples were subjected to cyclic strain for 7 days, they were trypsinized and resuspended in medium at a density of 2.0 × 10^6^ cells/ml. By using the collagen-based Cell Contraction Assay kit (Cell Biolabs, Inc.), we prepared a collagen lattice with bovine type I collagen at a concentration of 3.0 mg/ml. As indicated in the protocol of the kit, the collagen gel was polymerized in the presence of cells and incubated for two days to allow stresses to develop within the gel. Before the stress was released by detaching the gel from the culture dish, oxytocin (10 nM) was added to promote contraction as in SMC. Seven days after releasing the gels from the culture dish, pictures were taken and the surface area of the sample was measured using ImageJ.

### Statistical analysis

The statistical significance was assessed using Student’s t-test, F-test, or ANOVA followed by Fisher’s least significant difference (LSD). P-values below 0.05 were regarded as significant.
